# Methods for Natural and Synthetic Polymers Recovery from Textile Waste

**DOI:** 10.3390/polym14193939

**Published:** 2022-09-21

**Authors:** Daniela Simina Stefan, Magdalena Bosomoiu, Mircea Stefan

**Affiliations:** 1 Department of Analytical Chemistry and Environmental Engineering, Faculty of Chemical Engineering and Biotechnologies, University Politehnica of Bucharest, 1-7 Polizu Street, 011061 Bucharest, Romania; 2 Pharmacy Faculty, University Titu Maiorescu, 16, Ghe.Sincai Av, 040441 Bucharest, Romania

**Keywords:** textile waste valorization, environmental pollution, textile industry

## Abstract

Trends in the textile industry show a continuous increase in the production and sale of textile materials, which in turn generates a huge amount of discarded clothing every year. This has a negative impact on the environment, on one side, by consuming resources—some of them non-renewables (to produce synthetic polymers)—and on the other side, by polluting the environment through the emission of GHGs (greenhouse gases), the generation of microplastics, and the release of toxic chemicals in the environment (dyes, chemical reagents, etc.). When natural polymers (e.g., cellulose, protein fibers) are used for the manufacturing of clothes, the negative impact is transferred to soil pollution (e.g., by using pesticides, fertilizers). In addition, for the manufacture of clothes from natural fibers, large amounts of water are consumed for irrigation. According to the European Environment Agency (EEA), the consumption of clothing is expected to increase by 63%, from 62 million tonnes in 2019 to 102 million tonnes in 2030. The current article aims to review the latest technologies that are suitable for better disposal of large quantities of textile waste.

## 1. Introduction

Around 6 million tonnes of textiles are discarded every year in the European Union (EU), approximately 11 kg per person [1]. The textile industry is one of the most polluting, requiring large amounts of energy to produce synthetic fibers, and generally to produce clothes, consuming water and rejecting millions of liters of contaminated water every day, and contributing to greenhouse gas emissions [2]. In the EU, the textile industry has the fourth highest negative impact on the environment and climate change and the third highest negative impact for water and land use [1]. Water is mainly used for washing during the application of dyes. For instance, the presence of starch on natural fibers can block dye penetration into the fiber, as it is necessary to remove the starch before dyeing or printing [3]. It is estimated that about 200 L of water are used to produce 1 kg of textile materials [4]. The waters resulting from the production of textile materials are contaminated with organic compounds and metals such as Cr, As, Cu, and Zn [4,5].

Textile recycling aims to recover fibers, yarn, and fabrics, moving away from the traditional economy—which assumes manufacture, use, and disposal. The increase in demand and consequently in the production of textiles requires their valorization at the end of the life cycle in the context of the circular economy, thus keeping the materials in the loop as much as possible. The transition to a circular economy requires changes in production and consumption patterns.

To implement the circular economy, a stronger collaboration between textile manufacturers and recycling companies is needed, to collect information about the composition of textile materials, define methods for sorting these materials, and develop efficient methods of waste recycling and resource recovery.

Analyzing and modifying textile fabrication technologies to minimize the waste generated in the production steps is another major aspect that must be considered when speaking about the waste produced by the textile industry. The environmental impact may be reduced, for example, by avoiding unnecessary textile production, but not by transporting a large quantity of waste over long distances to be recycled. In addition, if the replaced method of production is not excessively polluting the environment, the overall gain is relatively small.

Three major fiber types are used in the textile industry: (1) natural, produced from natural resources such as: cotton (cellulosic fiber), wool, linen, and silk (protein based); (2) regenerated, derived from natural polymers, but requires treatments and processing; (3) synthetic, synthesized from petrochemical resources (non-renewable) such as: polyester, polyamide, acrylic fiber, and polypropylene [6]. Depending on the fiber type, different dyes are used: cellulose fibers are dyed using reactive dyes, direct dyes, naphthol dyes, and indigo dyes; protein fibers are dyed using acid dyes and lanaset dyes; synthetic fibers are dyed using disperse dyes, basic dyes, and direct dyes [4,7].

**Figure 1:**
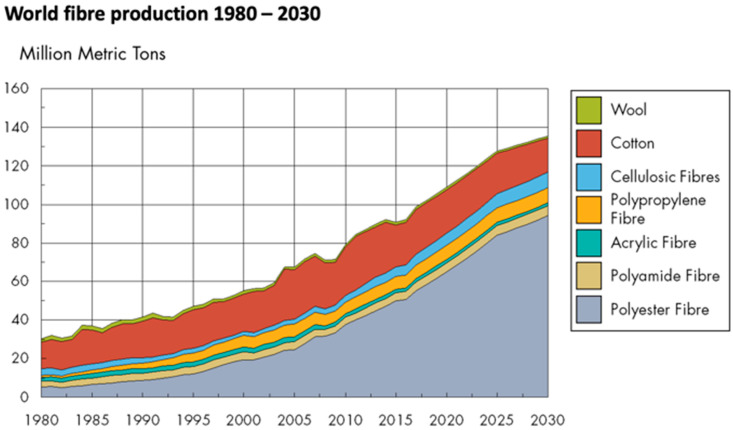
World fiber production for the period 1980–2030, reprinted with permission from Ref. [11].

The world fiber production has continuously increased over the last decades (Figure 1). The polyester fiber has the largest advance, followed by cellulosic fibers and polypropylene fibers. The production of cotton and wool has been almost constant in the last fifty years. Sixty-three percent of textile fibers are derived from petrochemicals [8]. The production of synthetic fibers has increased from about 47,400 thousand tons in 2010 to about 76,500 tons in 2019 [9]. There is no statistic on the percentage of polymers recovered from textile waste in recent years. This is because the textile industry and recycling companies do not yet have a clear protocol on the reuse of textile waste, as it is, in the case of household appliances. Each year in the European Union, around 2.1 million tonnes of post-consumer clothing and home textiles are separately collected for recycling or resale (38% of textiles placed on the EU market). The remaining 62% are thought to be discarded in mixed waste streams and go to landfill or incineration [1].

Based on data provided by Tecnon OrbiChem, it is estimated that by 2030, synthetic fibers will exceed 73% of total fiber production globally, with polyester accounting for 85% of this by 2030. This trend indicates that in the future, most of our clothing will be produced from fossil fuels, which contradicts the energy production trend, which is shifting towards green energy production [10].

Clothing is the most common article that people buy. The amount of textile waste is continuously increasing, due to an increased consumption related to the decrease in the price of clothing products, the decrease in the clothing quality, and the increase in the world population [12].

To improve the sustainability of the textile industry, the waste quantity must be minimized by increasing the population’s awareness of the consequences of their habits, increasing the quality of clothes, reusing them when the degree of wear is not high, or by transforming them into new products by repairing, upgrading, and remanufacturing (e.g., clothing that can be converted into blankets or carpets).

Disposal methods must include the recovery of the materials, energy, and chemical substances that were used in the production of textiles [13].

Schmutz and Som (2022) performed a quantitative study of the possible application of circular economy concepts in the Swiss textile industry [14]. In the case of Switzerland, large quantities of high-quality textile waste of known composition are being incinerated, in accordance with the traditional linear economy (produce, use, dispose).

Most of the clothing is made from a mixture of natural and synthetic polymers. The garments are often multicomponent, and therefore present a major recycling problem, as the fibers must be separated into individual components to enable effective recycling.

**Figure 2:**
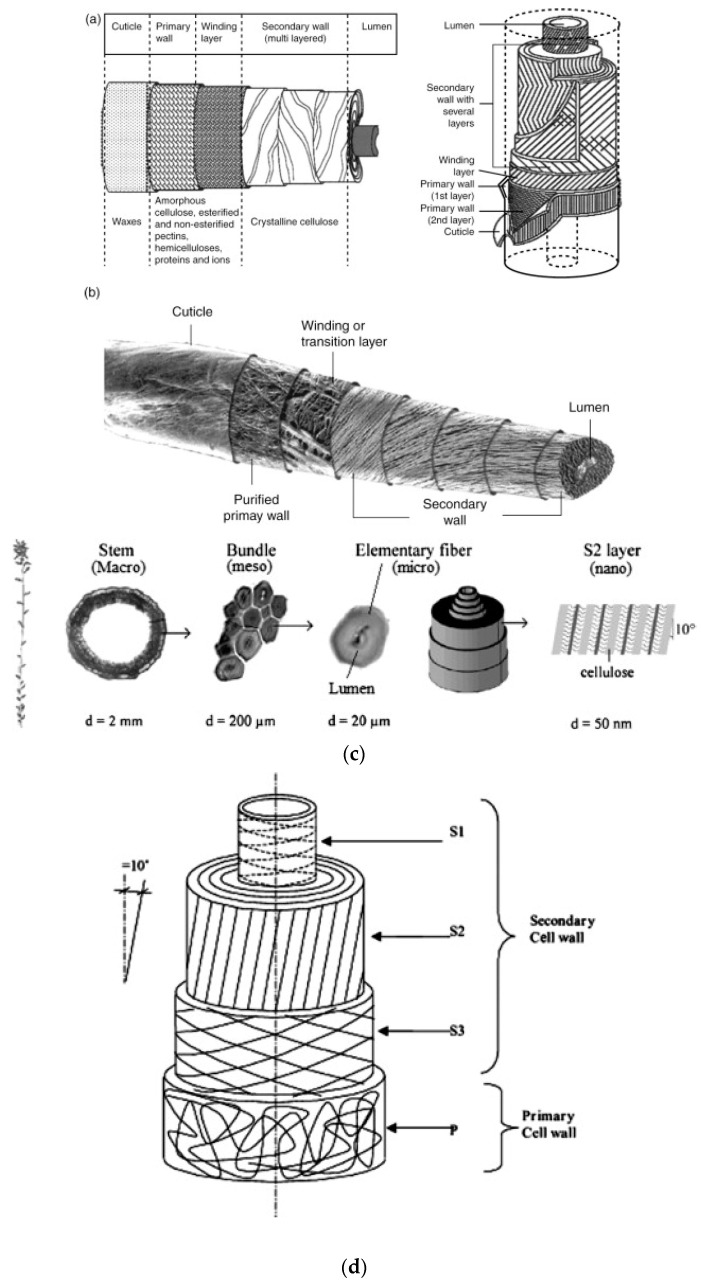
Schematic representation of natural fiber: (**a**) cross-section of cotton fiber. Typical components in dry, mature cotton fibers and composition of each layer, reprinted with permission from Ref. [16]. (**b**) Morphological model of cotton fiber, reprinted with permission from Ref. [16]. (**c**) Flax structure, reprinted with permission from Ref. [18]. (**d**) The micro-structure of a flax fiber cell, reprinted with permission from Ref. [19].

Cotton is a natural polymeric fiber extracted from seeds [15]. It has a multilayer structure of a non-cellulosic and cellulosic primary wall, cellulosic secondary wall, and a lumen (Figure 2). The primary wall is made of waxes, pectin, protein, hemicelluloses, and metal ions [16]. Recycling the cotton fibers leads to shorter cotton fibers of lower quality, which is the main obstacle to cotton recycling [17].

As can be seen in Figure 2c,d, flax fiber is a complex structure made from cellulose, hemicellulose, wax, lignin, and pectin, in varying proportions [17,20]. Cellulose, hemicellulose, and lignin are basic components, which determine the physical properties of the fibers.

Cotton production by itself has a negative impact on the environment, through the associated water consumption necessary for irrigation, use of pesticides, and soil erosion. Another disadvantage is the use of land for growing cotton crops, instead of agricultural products, which impacts the global food crisis. It was estimated that about 16% of global pesticide consumption and 3% of global irrigation water consumption are used for cotton crop cultivation [21].

**Figure 3:**
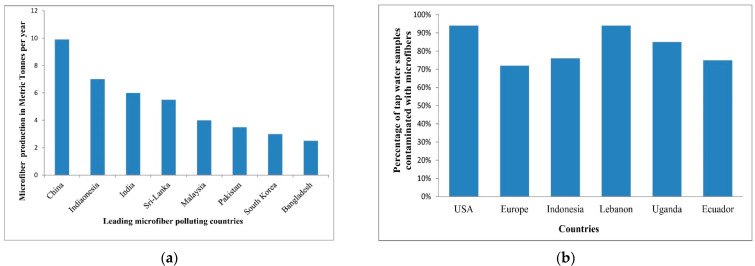
(**a**) Microfiber-generated pollution based on microplastic generation rate (2018); (**b**) microfiber contaminated tap water (2017), reprinted with permission from Ref. [23].

Synthetic fibers are polymers (such as polyester, nylon, acrylics, spandex) that have good durability, high moldability, and low cost and are not toxic in contact with the skin. However, they are non-biodegradable. During the clothing manufacture, and further on, while washing, plastic fragmentation takes place and generates so-called microplastics (particles with a diameter below 1 mm). These particles spread in the air, soil, and water streams, and accumulate in the bodies and tissues of living organisms entering the food chain (Figure 3). Among the countries generating microplastics, China is generating the highest quantity of microplastics because of its continuously developing economy. In Europe, over 70% of the tap water samples analyzed are contaminated with microplastics. Furthermore, microfibers can be retained on the surface, which are hazardous substances and can be passed on to the organisms that ingest them [22].

Although neglected for years, the problem of microplastics generated by the textile industry has started to gain a lot of interest from the scientific community [23,24,25,26,27]. Microorganisms located in the lower food chain are more at risk of suffering microplastic contamination [28]. The highest amount of microplastics is released in the first washes of clothes [1]. A single garment washing causes a loss of synthetic material of more than 1900 fibers per wash [29]. This fact has prompted washing machine manufacturers to consider installing microplastics filters [30,31] and textile manufacturers to elaborate lists of tips on how to reduce textile microplastics [32]. Both natural and synthetic fibers have been found in living organisms in seas and oceans. Fourier transform infrared (FTIR) analysis of fibers found in the sea urchin Paracentrotus lividus, in the Mediterranean Sea, revealed that 67% of the fibers were natural (cotton-based) and 33% synthetic polymers (polyester) [33]. This suggests that one of the fiber sources is laundry. A qualitative study of plastic presence in fish species in the Central Mediterranean Sea showed that about 97.1% of the plastic present in fish is fibers. Of this, 94.1% is microplastic, and only a small percentage (5.9%) is macroplastic [34]. FTIR analysis of samples from the southern Pacific Ocean (Chile and Peru) showed the presence of cotton and polyester fibers in marine species [35]. This suggests that water pollution with microplastics and natural microfibers is not a phenomenon that occurs only in the Mediterranean Sea, but a widespread one. Regarding the occurrence of synthetic microfibers in the marine environment, polypropylene was found to be the most common in water and sediments, followed by polyethylene in water, and polyester in water and sediments [36]. The sources of microplastics and the mechanisms of contamination of rivers and seas with synthetic and natural fibers are complex and not yet fully understood. One thing that is certain is their negative impact on living organisms.

Natural fibers provide good water absorption, while synthetic fibers provide strength and improved properties (e.g., elasticity).

**Figure 4:**
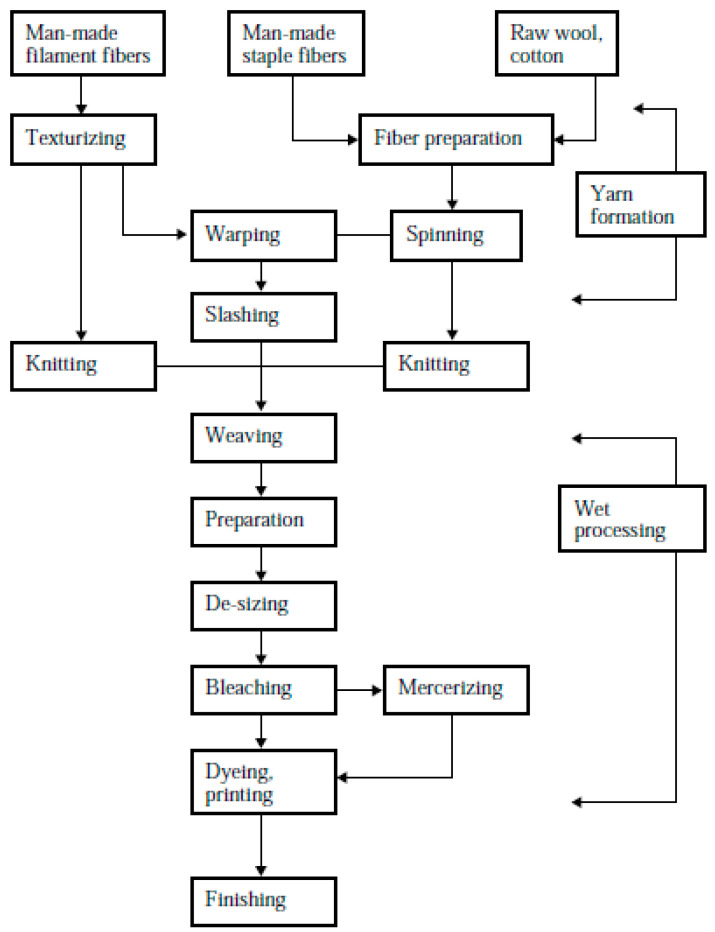
Steps in textile processing in a cotton mill, reprinted with permission from Ref. [3].

The steps followed to transform natural fibers into clothing are shown in Figure 4. Fibers go through several steps before being transformed into clothing. These steps include dyeing, knitting, printing, finishing, and so on [3].

The waste generated by the textile industry can be classified into two types: (a) waste generated during the fabrication steps, and (b) post-use waste. This article aims to review the methods applied so far, and future methods that can be used to more efficiently recover the resources contained in post-consumer textile waste. If recycling is applied, another aspect that must be considered is the composition of the waste generally made by mixing different types of yarns and dyes.

## 2. Methods for Textile Waste Removal or Recycling

To achieve a sustainable textile industry, the directions for the valorization of textile post-consumer waste are: (i) valorization as reusable goods for as long as possible; (ii) valorization as new products.

At the current moment, the recycling rate of textile materials is very low all over the world. According to EPA (United States Environmental Protection Agency), the recycling rate for all textile materials was around 15% in United States for the year 2018 [37].

### Landfill

**Figure 5:**
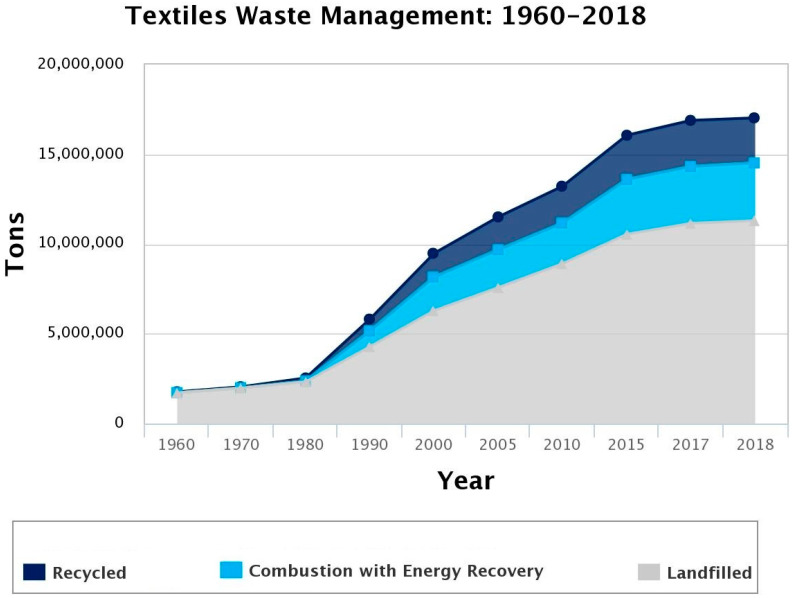
Trends of textile waste disposal for the period 1960–2018, reprinted with permission from Ref. [37].

Before 1980, generated textile waste was almost exclusively landfilled (Figure 5). After 1980, the quantity of generated textile waste was constantly increasing, and landfilling was the most used method for waste disposal (more than half of the waste is landfilled).

More than two-thirds of textiles go to landfills at the end of their use, and only around 15% is recycled [6].

In the conditions of a food shortage, a large area of agricultural land is occupied by the cultivation of crops designated for the fabrication of natural fibers. These crops also need water supply for growing, and the use of chemicals, to maintain a good-quality crop.

Landfilling raises a lot of problems related to land availability, greenhouse gas generation during decomposition [38], and leaching of toxic chemicals and dyes in the groundwater and soil. Although this solution presents many disadvantages, much textile waste is still disposed by landfilling.

**Figure 6:**
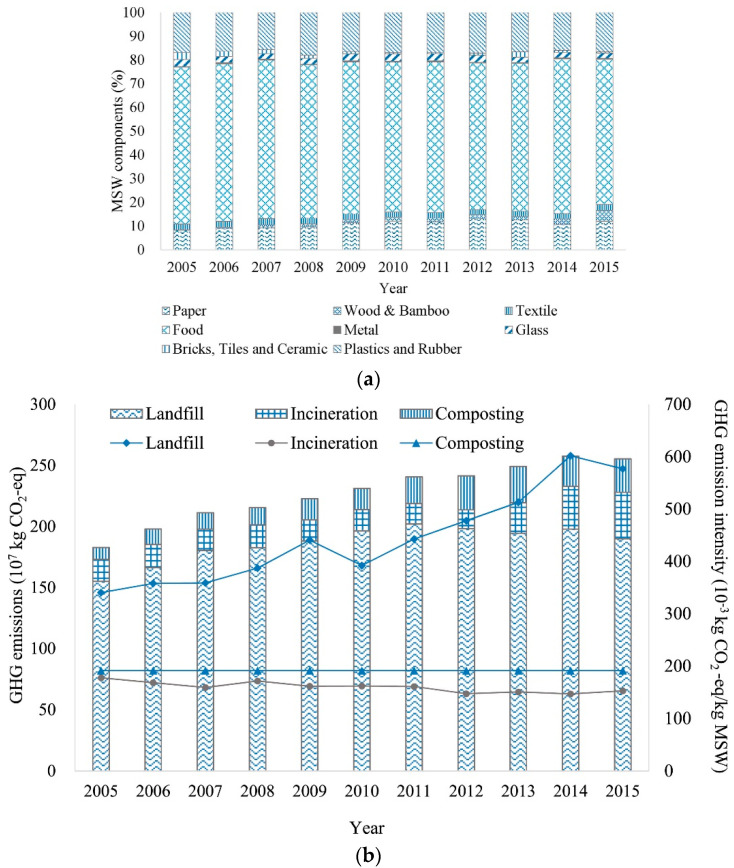
(**a**) Compositions of MSW (municipal solid waste) components in Shanghai (2005–2015); (**b**) GHG emissions and the intensity of three MSW disposal methods (2005–2015). The left axis used for the bar graph represents the GHG emissions, and the right axis used for the broken line graph represents the GHG emission intensity, reprinted with permission from Ref. [38].

Figure 6 shows the composition of municipal solid waste in Shanghai, along with the associated disposal methods and GHG (greenhouse gas) emissions. Non-renewable resources (petroleum) are used to produce synthetic textile materials. This contributes to the accumulation of non-biodegradable materials in the land. Landfill is followed by the combustion method, and recycling is about one-quarter of the disposal methods.

## 3. Combustion

**Figure 7:**
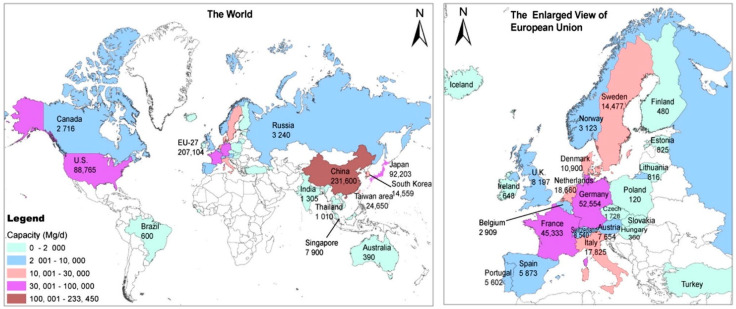
Global map of MSW incineration, reprinted with permission from Ref. [40].

The second most used method for textile waste disposal is combustion. When speaking about the combustion of wastes, the heating value, or the thermal energy stored in that waste, must firstly be evaluated [39]. Two parameters are employed to evaluate the heating value of municipal waste: higher heating value (HHV), which is the heat emitted during the complete combustion of 1 kg of waste, and lower heating value (LHV). The difference between the LHV and HHV of a combusted material is equivalent to the amount of latent heat of vaporization, which can be practically recovered in a secondary condenser. In addition, waste humidity plays a key role in the assessment of incineration conditions. It was found that adding textile waste and clothing to municipal waste improves its LHV value from 5000 kJ/kg to 9000 kJ/kg [40]. This method is mainly used in China, North America, and some European countries (Figure 7). Although incineration enables the reduction of the waste volumes in a short time, it generates hazardous chemicals such as benzene derivatives and polycyclic aromatic hydrocarbons (PAH), releasing large quantities of carbon dioxide. It was estimated that the combustion of one ton of waste textiles releases about 10 tons of CO_2_ into the atmosphere [41]. In addition, the contents of non-volatile heavy metals, such as Ba, Cr, Mn, and Ni, could significantly increase these emissions, by the presence of metallic accessories, as reported in the case of clinical waste incineration [42].

Recycling clothes is a complicated process, due to the mixing of different types of materials (natural and synthetic, textile and non–textile) in the same product (e.g., a cotton T-shirt contains labels and accessories that are made with synthetic materials; a pair of jeans is made from fibers of cotton and elastane, together with other parts such as zips and buttons). The separation of those materials is time consuming and laborious.

Sometimes, to facilitate the subsequent processing stage, it is necessary to cut the textile materials. The shredding process results in shorter, weaker fibers that cannot be reused to make clothes. To recycle the polymers (whether natural or synthetic) contained in the fabric, several chemical treatments are applied to separate them.

## 4. Chemical Treatment

One way to overcome the time-consuming separation of natural and synthetic fibers by manual selection is the use of a chemical treatment [43,44,45,46,47,48]. Waste jeans represent an important fraction of the total textile waste. They are a major source of cellulose. Natural cellulosic fibers are a renewable biomass source to produce higher-value products via cellulose hydrolysis. The cellulose contained by the cotton fibers has high crystallinity, being inefficient in the conversion to biofuels. Alkali pre-treatment (with NaOH or Na_2_CO_3_) at moderate temperatures transforms the crystalline structure of cellulose into an amorphous form, which is more easily biodegradable [49].

Palme et al. (2017) used a treatment with NaOH at temperatures ranging from 70 to 90 °C to separate cotton and PET (poly (ethylene terephthalate), polyester) from mixed textiles. In this step, the PET is degraded to terephthalic acid (TPA) and ethylene glycol (EG). Three product streams are generated from the process: cotton, TPA and the filtrate containing EG, and the process chemicals [43].

The separated materials can be further used to produce new chemical products. For example, Gholamzad et al. (2014) studied the production of ethanol from the cellulose recovered from a polyester-cotton textile material [44]. In previous studies, it was found that the addition of urea, thiourea, or a mixture of these two enhanced the dissolution of cellulose in an alkaline solution [45,46,47,48]. The alkali pre-treatment is followed by enzymatic hydrolysis, in the presence of cellulase and glucosidase. Alkali pre-treatment was found to improve the production of ethanol from the cellulosic part of a polyester-cotton blend [44].

Hasanzadeh et al. (2018) used Na_2_CO_3_ as the reagent for the alkali pre-treatment. The waste textile consisted of used jeans made of cotton and polyester with about 90% cellulose. The alkali-pre-treated textile waste was subjected to anaerobic digestion, enzymatic hydrolysis, and fermentation to produce biogas, sugars, and ethanol [49].

**Figure 8:**
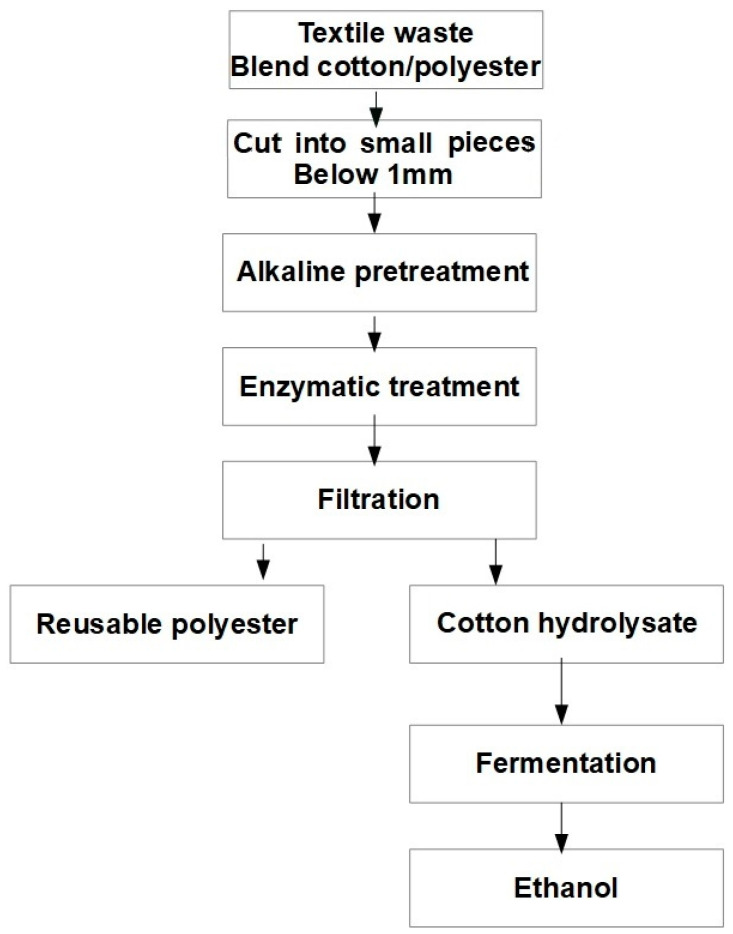
Schematic diagram of cotton-polyester waste blend valorization.

However, the studies presented above do not address the issue of dye removal prior to alkali treatment. The properties of recovered polyester as a value-added product were compared with untreated polyester, and it was found that its properties were only slightly modified compared to the original ones. In Figure 8, the steps for converting the cotton-polyester blended textile into ethanol and recovered polyester are shown.

It was shown that substituting conventional fuels by green fuels offers the advantages of disposal of waste material that is used for fuel production and reduction of GHG (greenhouse gases) emissions. One example is the use of cotton ginning wastes as an alternative energy source to replace part of the heavy fuel oil used for the thermal needs (up to 52%) of a textile plant located in northern Greece [50].

**Figure 9:**
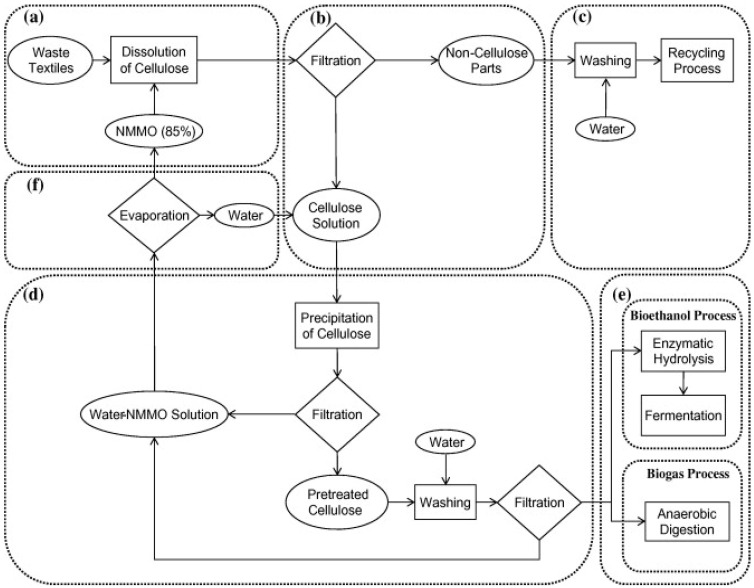
Cellulose separation from waste textiles using NMMO solution with recycling of solvent: (**a**)—mixing; (**b**)—filtration; (**c**)—washing; (**d**)—precipitation; (**e**)—enzymatic hydrolysis; (**f**)—evaporation. Reprinted with permission from Ref. [51].

Another possibility for valorization of the textile waste is the synthesis of different chemicals, after the recovery of natural or the synthetic polymers from the clothing material, using environmentally friendly solvents. Jeihanipour et al. (2010) developed a process for the separation of the cellulose, cotton, and viscose blended fibers (Figure 9) [51]. The recovered polymers were used in the synthesis of ethanol or biogas. The separation of the polyester/cotton fibers was made for a 50/50 mixture of polyester/cotton. In the first step, the cellulose was separated using an environmentally friendly cellulose solvent, N-methylmorpholine-N-oxide (NMMO). The cellulose was separated by precipitation, by adding water to the mixture solvent-cellulose. In this way, the recovered cellulose fibers are more accessible to the hydrolyzing process by enzymes or bacteria. To obtain the ethanol, the cellulose was hydrolyzed by cellulase enzymes, followed by fermentation, to produce the ethanol. The biogas was produced through fermentation of the cellulose.

The separation of the polyester/viscose blended textiles was made for materials having the proportion 40/60 polyester/viscose. The polyesters resulted in fibers after treatment with NMMO solvent.

Another method for textile waste valorization is the production of biogas. Jeihanipour et al. (2013) tested cotton/polyester and viscose/polyester fiber blends with no pre-treatment or milling [52]. The textile waste (jeans) was grounded and mixed with solvent, N-methylmorpholine-N-oxide (NMMO) to dissolve the cellulose. The steps employed for cellulose recovery were described in a previous study [51]. In the two-stage process, the lag phase was shorter than in the single-phase CSTR. Comparing treated and untreated jeans textiles wastes, the semi-continuous two-stage process can treat higher organic load rates.

The alkali and organic solvent treatment methods have several advantages compared with incineration:

allows fast and easy separation of natural and synthetic polymers from blended fibers;

the resulting cellulosic fibers are much shorter and therefore more degradable for further transformation in glucose, ethanol, and biogas;

the reaction conditions are relatively mild (temperature below 100 °C and atmospheric pressure).

Treatment with NMMO solvent also allows the recovery of the solvent and its immediate use.

Cellulose hydrolysis using concentrated acid (H_2_SO_4_) allows the production of hydrogen from cellulose hydrolysate via subsequent fermentative process [53]. Ouchi et al. (2010) developed a method consisting of acid treatment of an uncut textile piece, followed by mechanical stirring, filtering, washing, and drying, to produce cellulose powder [54]. The recovery of sulphate ion from the hydrolysate was made by anionic exchange. The conversion of a towel to glucose, and further to ethanol, was made by using microwave-assisted treatment of the towel impregnated in concentrated sulfuric acid [55].

To obtain a good conversion of recovered glucose in subsequent valuable products, the glucose yield in the hydrolysate must be sufficiently high. It has been reported that a single-step acid hydrolysis (using sulfuric acid) does not achieve a high glucose content. When using a two-step method, combining hydrolysis with concentrated and diluted sulfuric acid, it was possible to get 90% glucose yield [56].

Yousef et al. (2020) used the nitric acid treatment to recover the cellulose contained in jeans waste [57]. The dyes are removed together with the spent acid that is regenerated with activated carbon. Polyester was dissolved and separated from cotton, by using a green switchable hydrophilicity solvent. The polyester and the solvent were regenerated by adding CO_2_ to the solution. The solidified polyester is separated by filtration. The recycling rate of the technology was more than 96% for jeans waste containing 84% (wt.) cotton and 16% (wt.) polyester [57]. To select the leaching media among the strong acid reagents (HNO_3_ or H_2_SO_4_), preliminary tests showed that the nitric acid is leaching only the dye, while the sulfuric acid solubilizes all the components of the jeans waste [58,59].

The major drawback when using concentrated acid pre-treatment is the necessity of acid recovery. In addition, most of the studies are performed with textile waste that is pre-washed, dried, and cut, which cannot be done on a large scale.

Kuo et al. (2014) found that pre-treatment of waste textiles with ortho-phosphoric acid resulted in an improved rate of enzymatic hydrolysis, reducing the sugar yield [60]. This is because the acid dissolves the crystalline cellulose structure, which is resistant to enzymatic processes. When saccharification and fermentation steps are performed simultaneously, the ethanol concentration is higher, and the process takes place in one single reactor. Dyed textile waste did not show any inhibitory effect on the ethanol fermentation activity of *Zymomonas mobilis*. Phosphoric-acid-pre-treated cellulose has been proven to have a higher initial hydrolysis rate and glucose yield [61]. This method was applied for cotton textile waste and for mixtures of 40/60 polyester/cotton textile waste. Furthermore, the ortho-phosphoric acid is characterized by non-corrosivity and non-toxicity, being safer compared to NaOH, H_2_SO_4_, or HNO_3_ [62,63]. The authors optimized the hydrolysis conditions so that they could efficiently recover 100% of the polyester with a maximum sugar recovery of 79.2% (when using 85% phosphoric acid, 50 °C, 7 h, ratio of 1:15) [61].

Lopatina et al. (2021) used ionic liquids to prepare a cellulose-based ultrafiltration membrane from white cotton waste [64]. Zhong et al. (2020) used ionic liquid (IL) 1-butyl-3-methylimidazolium chloride ([Bmim]Cl) to extract wool keratin. The extracted keratin was used to prepare a keratin/polyacrylonitrile (PAN) composite nanofibrous membrane with good antibacterial effects and high moisture permeability [65]. This method was also applied for the separation of blended natural and synthetic polymers. The polyester-cotton blended textiles were treated with ionic liquid 1-allyl-3-methylimidazolium chloride (AmimCl). The recovered cellulose was transformed into transparent cellulose films and high-purity polyester [66]. It was found that the cellulosic fibers extracted from textile cotton waste have increased mechanical properties compared to cellulosic fibers produced from wood pulp. This is linked to a higher degree of polymerization of waste cotton [67]. The ionic liquid is recovered by washing the cellulose or keratin with water.

Another aspect that must be considered when developing a plan for textile recycling is the removal of dyes. Generally, the dyes used in the textile industry are synthetic organic colorants (e.g., azo dyes) with properties that make them resistant to destruction by conventional treatment methods. For cellulose fibers, the following types of dyes are used: reactive dyes, direct dyes, naphthol dyes, and indigo dyes; for protein fibers, acid dyes and Lanaset dyes are used. Dispersed dyes, basic dyes, and direct dyes are used to dye synthetic fibers [68]. The removal of dyes needs to be done before the recovery stage. Even small concentrations of dyes in the water receptor affects the environment, by their toxicity and by blocking both the penetration of light and oxygen transfer [69]. The dyes can be present in very low concentrations of 1 mg L^−1^ in the receptor waters, and they can enter the food chain through aquatic organisms [70]. Among the methods employed for the dyes’ removal, enzymatic degradation is more effective [71,72]. Other parameters that affect the stability of dye complexes are temperature and acidity. The results showed that yellow or orange chromium complex containing an aromatic carboxyl group could be easily destroyed by either of them. The destruction of more stable black chromium complex is achieved by both heating and acidification [73].

The sludge resulting after the treatment of textile waste contains Pb, Cd, Cr, and other toxic elements that can leach into the environment [3,74]. Several studies have tested the efficiency of vermicomposting to reduce the metal content of the sludge [75,76,77,78,79]. The tests showed that, for the Eudrilus eugeniae, the bioaccumulation preference was in the order: Zn ≥ Fe > Mn = Cu > Cr = Pb = Cd. In addition, vermicomposting can be applied to reduce the spread of fungal pathogens, inoculums, and so on [80]. The resulting compost is suitable for use as a soil supplement because the earthworms produce the humus essential in the crop growth. The main disadvantage of the composting is the long time needed to transform the waste in compost, although the introduction of earthworms accelerates the process. Abbas et al. (2013) applied composted waste textile (cotton), alone and in combination with a commercial fertilizer, on sunflower crop. The highest significant improvement in plant growth were obtained for soils treated with cotton waste compost and those treated with cotton waste compost plus commercial fertilizer [81].

Araujo et al. (2007) tested the use of composted solid sludge from a textile mill in the growing of soybeans and cowpeas. According to the authors, the sludge was not harmful for the plant’s growth. However, more studies are necessary because of the short time scale of this one (up to 63 days after plant emergence) [82].

**Figure 10:**
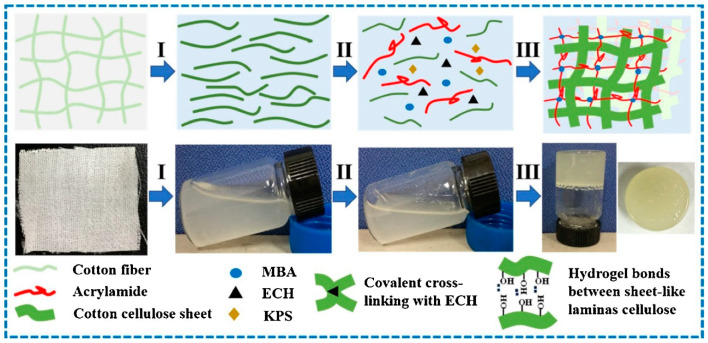
Synthesis of cellulose-based hydrogel: I—hydrolysis in NaOH, II—crosslinking, III—gelation process, reprinted with permission from Ref. [85].

The application of hydrogel-based materials has been extended in the last years to the removal of heavy metals from aqueous solutions [83,84,85]. One of the applications of the extracted cellulosic solutions via hydrolysis with NaOH is the adsorption of heavy metals from aqueous solutions [85]. A double network hydrogel is synthesized via crosslinking of natural polymer with synthetic polymer (polyacrylamide). The cellulosic hydrolysate is treated with KPS (potassium persulfate, which is the initiator) and crosslinked with MBA (N-methylene bis acrylamide). Different quantities of epichlorohydrin were added to this solution to obtain several hydrogel materials (Figure 10). These materials were tested in the retention of Cd, Cu, Pb, Zn, and Fe.

Another factor that increases the difficulty of clothes recycling is the presence of elastane. The elastane is a synthetic fiber synthesized from petroleum, and it has the advantage that it can be stretched to certain extent and returns to its shape when released. For that, it is widely used for sports clothing and pants to increase the functionalities of fabrics. Currently, most of the clothes containing elastane are not recycled because it is difficult to separate synthetic polymers from blends. Yin et al. (2014) developed a method for the selective separation of synthetic polymers from their blends and the recovery of the main polymer [86]. The separation of elastane from elastane/nylon blend clothes was made by a washing process at 220 °C using ethanol and water.

### 4.1. Thermal Treatment

Natural and synthetic polymers are a resource for recovering carbon. By using the tertiary recycling concept (turning waste into a completely new product), the carbon contained in the textile waste can be recovered as a new chemical substance. The pyrolysis process is the thermal decomposition of materials at high temperatures, in an inert atmosphere, and it involves changes in chemical composition. In this way, textile waste can be transformed after pyrolysis into three products: char, pyrolytic oil, and syngas. This method has been studied extensively in an attempt to find an efficient method of recycling textile waste [87,88,89,90,91,92,93,94,95,96,97,98,99,100,101,102,103,104,105]. The mechanisms and the kinetics of cotton pyrolysis are well known [106,107].

Another method studied for the reuse of textile waste is the synthesis of porous carbon through pyrolysis [108,109,110,111]. This method uses an additive: CaCO_3_, calcium acetate [111], or a natural source of CaCO_3_ (such as oyster shells) [112]. At high temperature, CaCO_3_ decomposes to CaO, releasing the CO_2_ and helping in the formation of the microporous carbon structure. The pyrolysis process follows the next steps: (a) raising the temperature of the material to be pyrolyzed using an external source; (b) initiation of pyrolysis reactions at high temperature; (c) release of volatile compounds; (d) formation of residues containing carbon.

**Figure 11:**
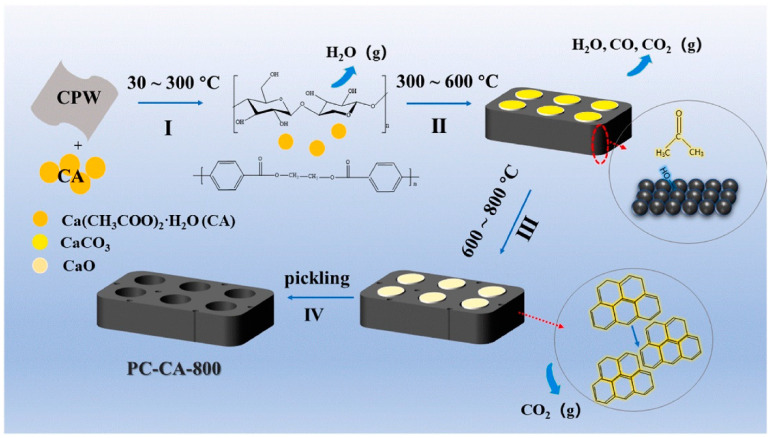
Schematic representation of carbon porous structure formation, at the pyrolysis of a cotton-polyester waste (CPW) in the presence of calcium acetate, reprinted with permission from Ref. [111].

By applying this method, Gu et al. (2022) synthesized porous carbon from a cotton/polyester textile with a high specific surface area (1106.63 m^2^/g), which was tested for the adsorption of tetracycline, a widely used antibiotic, which is a source of water contamination [111]. The steps involved in the porous carbon formation mechanisms are given in Figure 11 [111,113,114,115]:

(1) 30–300 °C: calcium acetate (CA) is dispersed on the surface of cotton polyester waste, and water contained by CPW is evaporated.
(2) 300–600 °C: CPW degradation to furans, sugars, ethylene, and aldehydes. At 400 °C, CA decomposes into acetone and CaCO_3_. Formation of aromatic structure through the cross-linking and cyclization reaction; construct preliminary carbon skeleton of porous carbon.
(3) 600–800 °C: CaCO_3_ decomposes into CaO and CO_2_, which helps in the formation of the mesoporous structure.
(4) CaO is washed by HCl solution, and porous carbon is obtained.

By changing the operating parameters (temperature, textile waste composition, residence time), the composition of the pyrolysis gas (H_2_ and CO) can be adjusted.

Another product that can be used after the pyrolysis is pyro-oil. Its composition can be improved by using catalysts (catalytic pyrolysis) [98]. ZnO was found to have the highest catalytic effect in the pyrolysis of mixed textiles, while Fe_2_O_3_ had the highest catalytic effect in the process of char gasification. The waste textiles were cut into very small pieces, with a particle size range of 0.8–1.2 mm, and the catalyst (single or composite) was loaded onto waste textiles. The major disadvantage of this method is the difficulty in pre-processing large quantities of textile materials to be pyrolyzed [89,98].

Comparing non-catalytic and catalytic pyrolysis, to minimize the production of benzene derivatives and polycyclic aromatic hydrocarbons, the addition of CO_2_ leads to three-fold higher H_2_ and eight-fold higher CO production [89].

Pyrolysis of flax textile waste can be used to produce, in a first step, furans, and further, monocyclic aromatic hydrocarbons. Furans are produced by catalytic dehydration of cellulose and/or hemicellulose in biomass [97,116,117,118].

Wang et al. (2018) studied the mechanisms of the catalytic fast pyrolysis of cellulose, cellobiose, and glucose in the presence of the zeolite catalyst NaY to decrease the activation energies of cellulose, cellobiose, and glucose [97]. The contents of furans after cellulose, cellobiose, and glucose pyrolysis in the presence of NaY were more than doubled (from 17.48%, 18.79%, and 28.77% to 46.71%, 52.11%, and 67.81%, respectively).

Catalytic degradation of flax waste (FW) to generate furans followed by Diels–Alder transformation to monocyclic aromatic hydrocarbons over USY zeolite (Si/Al molar ratios of 5.3 and 11.0) resulted in a 5.5-fold increase in furans production [118]. Three types of waste were tested: polyethylene (PE), flax waste (FW), and polypropylene (PP). PE co-fed with FW yielded almost two times higher aromatic hydrocarbons than PP. The selectivity to aromatic hydrocarbons was 81.6% for a mixture of 20% PE co-fed with 80% FW, in which benzene, toluene, and xylenes (BTX) were predominant products, with a maximum selectivity of 68%.

Textile waste has low calorific value. One method to remedy this is co-pyrolysis of textile dyeing sludge with high-calorific plastic waste (medical plastic wastes from syringes, medical bottles). Polyolefin plastics with high calorific value and low ash content have an optimum pyrolysis temperature in the range of 400–550 °C [88].

**Figure 12:**
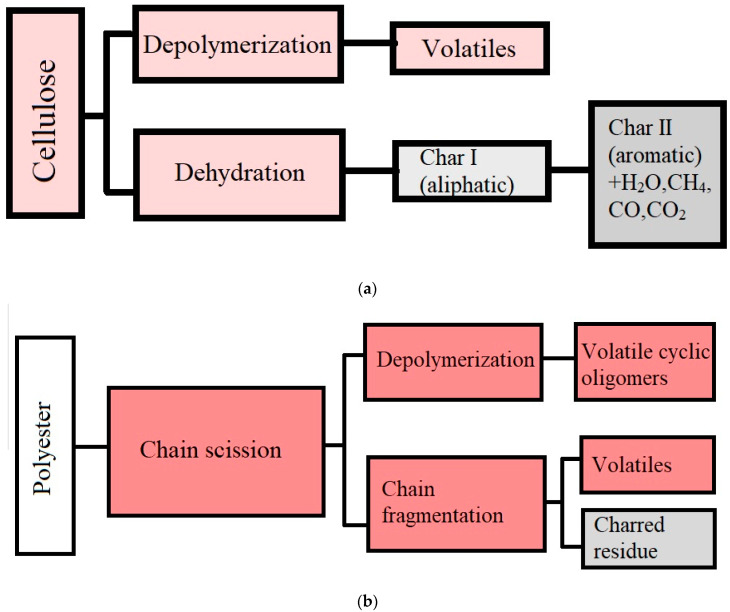
Mechanisms of cellulose (**a**) and polyester (**b**) degradation during thermal decomposition, adapted with permission from Ref. [108].

Alongi et al. (2013) studied the synthesis of char from cotton, poly(ethylene terephthalate), and their blends by thermal oxidative degradation. The researchers proposed two mechanisms for the transformation of cotton and polyester, respectively (Figure 12) [108]. In the case of cellulose, its thermal decomposition takes place at temperatures ranging from 300 to 400 °C through two concurrent steps: depolymerization and dehydration. The aliphatic structures generated after dehydration are further transformed into aromatic structures at about 400–600 °C. In a first step, the polyester undergoes chain scission, and at about 400–500 °C, it can either undergo depolymerization or decompose into char. The obtained char is thermally stable up to temperatures of 800 °C.

The resulting product can be used either as an adsorbent material or as an additive to improve the properties of clothing. Cay et al. (2020) investigated the use of textile waste-based biochar as additives, to improve clothing performance by changing the surface properties of textile materials. Cotton, cotton/polyester, and acrylic textile wastes were carbonized at low temperature, and the resulting products were applied to cotton by a printing method. This gives a slight hydrophobicity to the material, by increasing the water spreading speed and radius. Therefore, the acceleration of moisture transfer and drying has been improved. In addition, it was found that the resulting material has odor-masking properties [110].

Torrefaction has also been studied as an alternative method to produce quality fuels from textile waste [119,120,121]. Torrefaction is a mild form of pyrolysis at temperatures in the range of 200–320 °C. The resulting product has a lower moisture content and a higher heating value. The fuel characteristics of obtained biochar are comparable to those of coal [121]. The torrefaction temperature has a significant effect on biochar yield, while the fiber type influences the energy densification ratio (ratio of the HHV of biochar to the HHV of raw waste textile) [119]. Different types of textile waste (natural, synthetic, and their blends: cotton/polyester, acrylic/wool, acrylic/polyester, acrylic/viscose) were torrefied at temperatures in the range of 300–400 °C. The resulting material has low ash and sulphur content [119]. The 100% polyester fiber has high thermal stability, and is therefore not suitable for the torrefaction process. Torrefaction of polyester-containing blends, however, produced energy-dense biochar. Torrefied materials obtained from acrylic waste materials have properties similar to bituminous coal, while cellulosic polyester-based biochars have a similar structure to lignite. The biochar obtained from acrylic materials has a high nitrogen content (12–23 wt.%), which makes it unusable as a fuel.

Hydrothermal carbonization is also a thermochemical process used to pre-treat waste with high moisture content, under hot compressed water. Subsequent treatment of the obtained product is required to achieve the final product (e.g., activated carbon) [122,123,124,125,126,127,128,129]. Compared to a chemical treatment that uses strong reactants, in hydrothermal carbonization, under the effect of the pressurized water, the polymers contained in the textile waste are hydrolyzed and degraded into smaller molecules [130].

Another advantage of hydrothermal carbonization as a pre-treatment for textile waste, besides allowing the transformation of a waste into a valuable product, is the improvement of activation performance of the textile waste. This, in turn, reduces the quantity of activation agent (FeCl_3_, ZnCl_2_, KOH, CO_2_), which is needed in a subsequent step. Among the cotton, polyester, and cotton/polyester blends subjected to hydrothermal carbonization, the cotton-based ZnCl_2_-activated carbon exhibited the highest surface area (1906 m^2^/g), and an andoxytetracycline adsorption capacity of 621.2 mg/g, whereas the polyester-based adsorbent had a lower surface area (554 m^2^/g) and lower oxytetracycline adsorption performance [122]. FeCl_3_ was found to lower the initial processing temperature, catalyze dehydration and decarboxylation, and facilitate the formation of furfural derivatives, which are subsequently transformed into lignin structure [125].

Hydrothermal carbonization applied to solid waste containing textile waste has also been investigated. Wood, paper, and food have been used to perform simple hydrothermal carbonization or co-hydrothermal carbonization [124]. Synergistic effects have been reported during co-hydrothermal carbonization: negative synergistic effects between textile, wood, and paper waste; positive synergistic effects between textile and food waste. Increasing the oxygen content of hydrochars decreased the HHV.

Co-hydrothermal carbonization of cotton textile waste and polyvinyl chloride waste (PVC) was carried out to recover the energy contained in the waste as an alternative solid fuel. During the thermal treatment of PVC, HCl is generated from the dechlorination process. HCl release allows the formation of the porous structure in the hydrochars [127].

When using hydrochloric acid, it is possible to extract the cellulose in its crystalline form [126,131]. Sheng et al. (2018) studied the extraction of microcrystalline cellulose by the hydrothermal method and compared the extracted product with a commercial one (Avicel PH101 microcrystalline cellulose). The microcrystalline cellulose was extracted under hydrothermal conditions, in the presence of HCl: solid–liquid ratio 1:30, HCl concentration 0.6 mol/L, 150 °C [126].

Waste cotton textiles were subjected to hydrothermal pre-treatment at 240 °C–340 °C, to obtain bio-crude oil and biochar [129]. The optimum conditions (320 °C for 60 min) allowed the highest bio-crude oil fraction of 23.3%. The obtained biochar has a rich carbon content (76.86%) and low oxygen content, and it can be used as an alternative fuel. Furthermore, the biochar was evaluated as an electrocatalytic material with good results (the biochar enhanced the conductivity of the electrode and improved the electrochemical surface area, which could speed up charge transfer rate).

### 4.2. Enzymatic separation

Compared to the other methods presented above, enzymatic degradation allows the degradation process to operate under mild conditions [132,133,134,135]. Furthermore, enzymes are biodegradable, and therefore harmful chemicals are replaced by materials that are easier to dispose of. Enzymes act as catalysts and, at the end of the process, can be reused. Enzymatic degradation is specific to natural polymers, allowing the separation of natural and synthetic polymers.

The separation of wool fibers from mixed wool and polyester fabrics by enzymatic digestion allowed the recovery of keratin hydrolysate (obtained from wool) and polyester [132]. Analysis of polyester by scanning electron microscopy showed that the enzymatic treatment had no significant change on the quality of recovered polyester fibers. Enzymatic degradation can also be applied to blends of several natural fibers with polyester. For that, two types of enzymes are used: protease, for the extraction of amino acids from wool components, and cellulases, for the recovery of glucose from cotton fibers. The glucose resulting from the cotton fibers is subsequently converted into ethanol, by fermentation with Saccharomyces cerevisiae [133].

A summary of the advantages and disadvantages of the methods used to dispose of textile waste is presented in Table 1.

## 5. Conclusions

This study has provided insights into the current methods available for treating post-consumer textile waste. The current situation of post-consumer textile waste could be improved, as large quantities of textile waste of good quality and well-known composition are going to be incinerated, or worse, landfilled. This can be remedied by better collaboration between the textile manufacturers and recycling companies, to better sort textile waste and collect large quantities of waste together. Among the different types of fibers, cotton and other cellulosic materials release more fiber fragments during the washing process than synthetic fabrics. However, synthetic fibers are considered a bigger threat to the environment because they have an extremely low biodegradation rate. Most textile products are dyed with various synthetic colorants, including reactive dyes, or coated with various chemicals to provide new functionalities (repeatedly stretching; water repellence; resistance to deterioration by body oils, perspiration, lotions, or detergents). The presence of these chemicals complicates the degradation of both natural and synthetic fibers. Several recycling methods have been shown to have advantages in transforming textile waste (blend of natural and synthetic polymers or not) into valuable new products. These methods are not closed-loop recycling methods (i.e., textile to textile); they are open-loop recycling methods (i.e., conversion of textile waste into glucose, ethanol, biochar). However, these methods are tested at a laboratory scale on small quantities that are mostly cut textiles. Another problem that arises when applying these methods is the necessity of previous dye removal. Chemical hydrolysis allows the recovery of both natural and synthetic polymers, mixed or not, and their transformation into new products. However, this is a slow process and uses highly corrosive reagents. Particularly attractive is the enzymatic degradation that allows the recovery of polymers from blend fibers under mild conditions and using biodegradable media (enzymes). In addition, the thermal transformation of textile waste could be a better method for the disposal of textile waste, which allows obtaining better quality fuels or new products that can be used as adsorbents. The main advantages of thermal methods are the removal of large quantities of textile waste with variable composition in a relatively short time. There is a lack of data on the actual application of all the presented methods in textile waste recycling, indicating that they are generally applied on a small scale. The economic impact of using one method or the other should be also assessed, to better decide which of these methods is more appropriate.

## Figures and Tables

**Table 1:** Main methods for the treatment of textile waste.

Process	Advantages	Disadvantages	References
Combustion	- applied to all type of textile wastes - does not require a previous separation of textile wastes - fast disposal of waste	- supplementary consumption of combustible for incineration - influenced by the degree of humidity of the waste - the resulting ash is contaminated with heavy metals from the coloring dyes and from the accessories - generates a large quantity of CO_2_ and atmospheric pollutants - generates ashes	[39,40,41,42]
Chemical hydrolysis	- applied for blends of different types - allows the treatment of blended polymers (natural and synthetic) - the extracted natural polymer has much shorter fibers, and is more easily transformed in other chemicals: glucose, ethanol, biogas - the properties of recovered polyester are comparable with those of a fresh material - if an environmentally friendly solvent (NMMO solvent) is used, the additional pollution caused by its use is avoided	In the case of NaOH or acids: - the process needs high quantities of water to wash the intermediary products. - relatively slow; it needs time to air-dry the resulting product for 1 day - highly corrosive reagents - for all type of reagents: - the additional chemical reagents’ recovery is necessary for reuse - if the textile composition varies significantly, the hydrolysis conditions (temperature, reagent concentration, time) need to be reconsidered.	[45,46,47,48,53,54,55,56,57,60]
Pyrolysis Co-pyrolysis Torrefaction Hydrothermal carbonization	- allows the disposal of large quantities of waste in relatively short time - applied to all type of textile wastes - the textile waste is transformed into a valuable product	- consumption of combustible - generates volatile substances	[87,88,89,90,91,92,93,94,95,96,97,98,99,100,101,102,103,104,105,106,107,108]
Landfilling		- land availability - greenhouse gas generation during decomposition - leaching of toxic chemicals and dyes in the groundwater and soil	[6,38]

## Data Availability

Not applicable.
